# The growth of *Molecular Brain*: impact factor is coming in 2013

**DOI:** 10.1186/1756-6606-5-37

**Published:** 2012-10-11

**Authors:** Tim Bliss, Bong-Kiun Kaang, Min Zhuo

**Affiliations:** 1 National Institute for Medical Research, London, NW7 1AA, Mill Hill, UK; 2 Seoul National University, 599 Gwanangno Gwanak-gu, Seoul 151-747, Korea; 3 University of Toronto, 1 King’s College Circle, Toronto, M5S 1A8, Canada

## 

We are pleased to announce that *Molecular Brain* has been accepted by Thomson Reuters for tracking and is due to receive its first official Impact Factor in June 2013.

We would like to thank our distinguished international editorial board for their efforts on behalf of the journal, and our publishers, BioMed Central, for their in-house contribution to the speed and efficiency with which manuscripts are processed. Most important of all, of course, are our authors and reviewers, and to them we extend our particular thanks.

Since it was launched in 2008, as the official journal of the Association for the study of Neurons and Diseases (AND), *Molecular Brain* has published over 175 articles covering a wide range of topics in basic and clinical molecular neurosciences. A rapid review process ensures that papers are published in a timely manner. Our average time from submission to the first decision is 24 days, even under our stringent peer review process. As an open access journal, all papers are made immediately and freely available and articles are being accessed more than ever, with an average of 18,000 accesses per month so far in 2012, and a record of 21,000 accesses in May 2012. Links to publications are now listed on the *Molecular Brain* Twitter account [1]. Authors can also access statistics about their article, such as how many times it has been accessed and discussed on social media websites. These statistics can be accessed by selecting ‘About this article’ in the right hand column of each article. In addition, BioMed Central is working to facilitate more mobile access to content by publishing articles in ePUB format, which can be easily downloaded and viewed on any eReader.

Each year the Molecular Brain Award is made to the corresponding author whose paper has received the most citations. This year the award was presented to Dr. Michael Schlossmacher (University of Ottawa) at the annual meeting of AND in Montreal (Figure 1).

**Figure 1:**
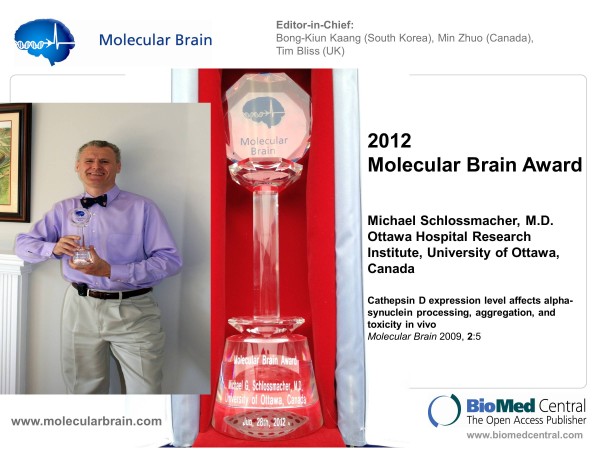
**Molecular Brain Award 2012 presented to Dr Michael Schlossmacher**.

A series of reviews on Molecules of Memory [2] is currently being published, and will be followed by another series of reviews on Molecules of Neural Disease.

With the help of its readers and authors we look forward to the continuing growth of *Molecular Brain*.

## Author details

^1^National Institute for Medical Research, London NW7 1AA, Mill Hill, UK.^2^Seoul National University, 599 Gwanangno Gwanak-gu, Seoul 151-747, Korea. ^3^University of Toronto, 1 King’s College Circle, Toronto M5S 1A8, Canada.

## References

[B1] Molecular Brain Twitterhttp://twitter.com/molecularbrain

[B2] Molecules of Memory serieshttp://www.molecularbrain.com/series/molecules of memory

